# Activity limitation and disability due to pain in back and extremities in rural population: A community-based study during a period of twelve months in rural Gadchiroli, India

**DOI:** 10.7189/jogh.11.12003

**Published:** 2021-11-27

**Authors:** Anand A Bang, Shekhar Y Bhojraj, Mahesh Deshmukh, Sameer Kalkotwar, Vinay R Joshi, Tushar Yarmal, Yogesh Kalkonde, Abhay T Bang

**Affiliations:** 1 Society for Education, Action and Research in Community Health (SEARCH), Gadchiroli, Maharashtra, India; 2 Spine Foundation, Mumbai, Maharashtra, India; 3 Hinduja Hospital and Research Center, Mumbai, Maharashtra, India; 4 Naraindas Morbai Budhrani Trust, Mumbai, Maharashtra, India

## Abstract

**Background:**

Population based estimates of the extent of the activity limitation due to back pain and disability due to musculoskeletal pains are lacking from rural India. We estimated this burden as a) extent of activity limitation due to back pain, b) disability due to musculoskeletal pains, c) grading of the limitation of each activity due to back and musculoskeletal pain in the rural adult population in Gadchiroli, India.

**Methods:**

This population-based, cross-sectional study was conducted in two villages randomly selected from a cluster of 7 eligible villages in Gadchiroli district of India. All adults ≥20 years in these villages were surveyed by the trained community health workers in January 2010. Disability due to back pain was evaluated using newly developed questionnaire for women and men which assessed limitations in the gender-specific daily household and occupational activities in a rural area. Disability due to pain in extremities was assessed using the Health Assessment Questionnaire (HAQ).

**Results:**

The total population of the two villages was 3735 out of which 2535 (67.9%) were adults ≥20 years of age and were eligible to participate in the study. Of these, 2259 (89%) were interviewed and 1247 participants (55%) had any pain on the day of the survey. Activity limitation questionnaire was filled for 716 (91.4%) out of 783 patients with back pain. HAQ scale was filled for 524 (85.2%) out of 615 patients with pain in extremities. Among men with back pain, respectively 11%, 19%, 60% and 11% had no, mild, moderate to severe difficulty or were completely unable to perform agrarian work, while among women, respectively 6%, 20%, 69% and 4% had no, mild, moderate to severe difficulty or were completely unable to perform household activities. Based on the HAQ score, respectively 1%, 67%, 18% and 14% of the participants had no, mild, moderate to severe disability or were completely unable to perform the activities.

**Conclusions:**

This community-based study in rural Gadchiroli demonstrates significant mild to moderate disability and activity limitation, due to pain in back and extremities in a population involved in hard manual work, especially agricultural and underlines the need to address the problem through appropriate interventions. The study also employs for the first time an indigenously developed questionnaire to identify activity limitation due to back pain, and demonstrates the method as well as the questionnaire.

Back pain (BP) and musculoskeletal pain (MSP) are the commonest form of chronic pain, causing disability and health care expenditure world over [1-4]. Additionally, low back pain (LBP) is the one of the most important cause of activity limitation in both men and women [5] as well as the second most frequent reason, after upper respiratory infections, for physician visits [6,7]. As heavy manual labor is a known risk factor for back and musculoskeletal pain [8], agrarian rural communities are at a high risk of BP and MSP [9-11] as well as disability and activity limitation.

The Health Assessment Questionnaire (HAQ) is a standardized tool for measuring disability due to musculoskeletal pains and has been used in Indian setting [12]. But disability and limitation to activities of daily living (ADL) due to low back pain are difficult to assess; and are measured by patient perceived responses in a questionnaire, either written or interview based. The two most commonly used LBP disability questionnaires, Roland-Morris (RM) and Oswestry, could not be used with the rural population in Gadchiroli as these Scales were developed in Europe, North America or Australia and consisted of inquiries regarding climbing stairs, driving and similar other activities inappropriate to rural Indian setting. Consequently, there was absence of culture specific questionnaire to measure disability due to low back pain in a rural population of India.

Hence population-based data from rural India on the burden of disability due to back pain and musculoskeletal pains is significantly lacking, limiting the possibility of developing appropriate interventions [12]. Therefore, in this study we aimed to develop an activity limitation questionnaire and estimate the activity limitation due to low back pain and disability due to musculoskeletal pains in the adult population in rural Gadchiroli in Central India.

## METHODS

### Study setting, design and sample size

This study of activity limitation due to pain in back and disability due to musculoskeletal pains was nested in a population-based, cross-sectional, interview based survey of the prevalence of PBE in rural Gadchiroli. The study setting, study design, detail method of sample size calculation, method of village selection including the eligibility criteria are described in detail [13].

### Designing local questionnaire for the assessment of activity limitation due to pain in back

The disability scales available in the western literature for back pain such as the Oswestry Disability Scale (ODI), the Rolland Morris (RM) Scale, WHO Health Assessment Questionnaire were reviewed and found to be not applicable to the community from rural Gadchiroli, and most probably to population across Rural India. Some activities of daily living mentioned in the western scales were rare in rural community such as climbing steps inside the house. If these activities are removed from the available disability Scales, then the standardized scoring system of these Scales distorts and the score thus obtained does not hold true. Also, Scales like ODI were designed for a population which can self-administer the scale which was not possible considering the low literacy level of our population. Additionally, these Scales were considered too lengthy to be administered in the field. Lastly, in the rural community, the activities of males and females can differ significantly. Hence it was deemed inappropriate to use a single disability scale for both the genders. To test these observations we translated RM scale and ODI in Marathi, the vernacular local language. These questionnaires were distributed to the focus groups of male and female community health workers numbering 60 from the local rural community and discussed with in depth. Their feedback matched with our above observations.

Therefore, we decided to develop a new ‘Rural Indian Activity Limitation Questionnaire’ (RIALQ) to assess the activities of daily living that may be affected by back pain in rural Gadchiroli. We applied the following principles while developing this questionnaire; 1) there should be participation of the local community, taking into account their daily activities, understanding which of their activities are limited due to back pain, and what according to them are the important activities amongst the limited ones; 2) the list of activities should be comprehensive and representative of different postures associated with the use of back but not too long making it impossible to use in field and that 3) develop gender specific questionnaires reflecting their respective daily activities. Based on these, we decided to include maximum 11 questions in the questionnaire which in our experience would ensure feasibility of administration without compromising on the quality.

RIALQ was developed through several steps. First, Focus Group Discussion (FGDs) were conducted with the community health workers (CHWs), separately with males and females. The CHWs were asked to make a free list all the activities limited due to back pain. A total 30 activities for males and females were identified. Second, the CHWs were individually asked to rank the selected activities by scoring on a scale of 0-10, and were advised to score higher for the activities which were done more frequently and / or were considered more important. Finally, the CHWs were asked to score which activity they would want to be treated on the highest priority if they were unable to perform the same due to back pain, reflecting the intervention priorities. The mean of the score by the CHW for each of the activities were calculated. The activities were classified into different key postures and the activities which topped the list from each of the postural group were picked up giving the final list of eleven activities for women and men each.

RIALQ was tested by administering to a conveniently selected sample of individuals in the rural clinic at Society for Education, Action and Research in Community Health (SEARCH) and in the villages by the field supervisors. Accordingly, the language of the various questions in the questionnaire was modified and the final questionnaire was developed. A simple scoring system of no difficulty (0), mild (1) and moderate difficulty (2) and cannot do the activity at all (3) was applied to RIALQ making 33 as the highest score for any individual signifying maximum activity limitations.

For musculoskeletal pains, the standard HAQ with standardized scoring system with 3 as the highest disability score for each of the 12 questions was used, making 36 as the highest score signifying maximum disability.

### Questionnaires

The HAQ questionnaire asked the participants whether they had no difficulty, mild difficulty, moderate to severe difficulty in doing certain activities or could not do the said activity at all. The activities assessed were wearing clothes, sleeping and getting up from wooden bed, lifting a full glass or cup to mouth, walking on plain ground, bathing and wiping body dry, squatting to defecate or sitting on the floor folding legs, bending down to lift things, using the tap, boarding and getting down from bus or any other vehicle, walking a distance of 3 km, shopping in vegetable market or grocery and climbing few steps.

The RIALQ used in men assessed the level of difficulty felt in performing various activities in the same way. The activities assessed were performing agrarian tasks such as ploughing or harvesting, cutting wood, lifting heavy things by bending or carrying and lifting on head, squatting for or getting up after defecation, travelling by bus, sitting upright or straight, standing for long time, sitting for long time, regular walking, walking farmlands or climbing up riverbeds (whether it is painful and needs support of stick or another person) and how was the sleep.

The RIALQ used in women assessed the level of difficulty felt in performing various activities as performing agrarian tasks such as sowing, harvesting, cutting of grass, household tasks as sweeping, cooking and washing clothes, drawing water from well or bore well, lifting heavy things by bending or carrying and lifting on head (items such as wooden logs, grass and water pots), squatting for or getting up after defecation, travelling by bus, sitting upright or straight, standing for long time, sitting for long time, regular walking, walking farmlands or climbing up riverbeds (whether it is painful and needs support of stick or another person) and how was the sleep.

### Training, quality control and data collection

All the field supervisors were trained in interviewing the participant using RIALQ in case of pain in back and HAQ for pain in extremities. Data were collected from 1 January 2010 to 25 January 2010 during which information about the immediate past 12 months from January 2009 to January 2010 was recorded, details of which are described in detail in the previous paper [13]. The patients with pain in the back and extremities (PBE) were identified during a cross-survey by the CHWs and were referred to a village clinic organized in the first week of February 2010, staffed by a team of spine surgeons and rheumatologists. Trained field supervisors’ interviewed these individuals and filled the HAQ and RIALQ.

### Statistical methods

A database was constructed for data entry using FOX PRO Version 2.0. The data were double entered, validated and checked for inconsistencies. Descriptive statistics included mean, medians and ranges for continuous variables and proportions for categorical variables were estimated. Analyses were conducted using Stata 10.0 (State Corp, College Station, Texas, USA).

### Ethical approval

The research followed the tenets of the Declaration of Helsinki. Ethical approval for this nested study was granted as part of the main study, by the Institutional Ethical Committee of SEARCH formed according to the guidelines by the Indian Council for Medical Research. Consent was obtained first at the cluster level in the study villages 15 days before starting the survey. The community leaders (Village Council Leaders and members, school teacher and presidents of microfinance self-help groups) were explained the purpose and scope of the study including the benefits to the villagers (availability of referral care in SEARCH clinic and the care through a village clinic). Informed written consent in vernacular language in a standard format was obtained from individual participants after explaining the nature and benefits of the study. The benefits provided during the study included free consultation by spine surgeons and rheumatologists in a clinic conducted in the same village at a later date. For those who needed further evaluation, laboratory investigations, as well as imaging with Magnetic Resonance Imaging (MRI) and x-ray including transport were provided free of cost. For patients needing pharmacotherapy, and physiotherapy, these services were also provided free of cost and for those needing surgical interventions, such services were provided at significantly subsidized costs. The CHW discussed these benefits using a printed pamphlet.

## RESULTS

### The study population and its characteristics

**Figure 1:**
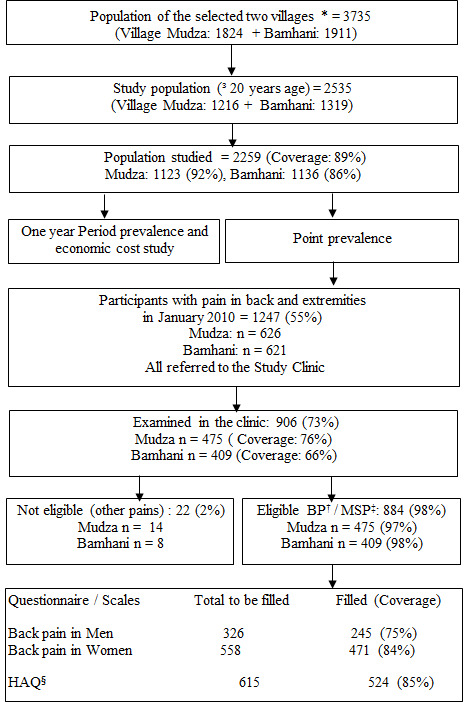
Study design flowchart. Legend: *population register 2010, †back pain, ‡musculoskeletal pain, §Health Assessment Questionnaire.

The total population of the two villages was 3735 out of which 2535 (67.9%) were adults ≥20 years of age and were eligible to participate in the study. Of these, 2259 (89%) were interviewed and 1247 participants (55%) had any pain on the day of the survey. They were referred to the village clinic conducted at a later date. Total 906 participants (73%) were followed up in the village clinic out of which 884 (98%) participants (326 males and 558 females) had back pain and 615 participants had pain in extremities. RIALQ was filled for 245 males (75%) and 471 females (81%), whereas HAQ was filled for 524 participants (85%) (**Figure 1**). The age, marital status caste and education distribution of the participants are presented in **Table 1**.

**Table 1:** Disability and functional activity limitation in rural Gadchiroli: Sociodemographic characteristic of the study population (n = 2259)

Characteristic	No.	%	
Population eligible (20 y and above) of total population	2259	89	
**In point prevalence:**				
Total Back & extremities pain	1247		55	
Total patient examined in clinic	906		73	
Total back & extremities pain patients	884		98	
**Distribution of 884 patients with pain in back and extremities:**				
**Gender:**				
Males	326		37	
Females	558		63	
**Marital status:**				
Unmarried	39		4	
Married	845		96	
**Caste:**				
Schedule caste	85		10	
Schedule tribes	129		15	
Other backward caste	670		76	
**Education (years):**				
Illiterate	464		52	
1-4	162		18	
5-7	83		9	
8-10	122		14	
11-12	43		5	
>12	10		1	
**Age (years):**				
20-30	121		14	
31-40	174		20	
41-50	233		26	
51-60	160		18	
>60	196		22	
Mean age (SD)	48.5 (15.3)			

SD – standard deviation

### Disability due to pain in extremities

The disability due to pain in extremities is shown in **Table 2**. Of the 524 participants whose HAQ was filled, a majority, ie, 75% and 80% had no difficulty in activities such as lifting a full glass or cup to mouth and using a tap respectively. The activities where the respondents more frequently reported moderate to severe difficulty were sleeping and getting up from wooden bed, climbing steps, walking three kilometres and squatting to defecate. Overall, no difficulty (zero), mild difficulty (>zero and ≤1), moderate to severe difficulty (>1 and ≤1.5) and complete inability (>1.5) to do an activity was identified in 1.3%, 67%, 17.6% and 14.1% of the participants respectively (**Table 3**).

**Table 2:** Disability due to musculoskeletal pains in rural Gadchiroli measured on the HAQ* scale (n = 524)

Question	No difficulty (%)	Mild difficulty (%)	Moderate to severe difficulty (%)	Cannot do at all (%)
	**0**	**1**	**2**	**3**
Wearing clothes	53	26	20	1
Sleeping and getting up from wooden bed	23	44	33	0
Lifting a full glass or cup to mouth	75	20	6	0
Walking on plain ground	42	36	21	1
Bathing and wiping body to dry	52	31	17	1
Squatting to defecate or sitting on the floor folding legs	26	38	30	6
Bending down to lift things	40	39	20	1
Using the tap	80	14	5	1
Boarding and getting down from bus or any other vehicle	34	41	23	2
Walking 3 km	22	39	31	8
Shopping in vegetable market or grocery	36	29	23	12
Climbing few steps	24	34	32	10

HAQ – Health Assessment Questionnaire

**Table 3:** Categorization of HAQ* score according to severity

Scale	Participants		Male participants		Female participants	
	**n**	**%**	**n**	**%**	**n**	**%**
No difficulty (0)	7	1.3	5	2.5	2	0.6
Mild difficulty (>0 &≤1)	351	67.0	136	67.7	215	66.6
Moderate (>1 &≤1.5)	92	17.6	38	18.9	54	16.7
Severe (>1.5)	74	14.1	22	10.9	52	16.1
Total forms filled	524	100.0	201	100.0	323	100.0
Mean (SD)	0.86 (0.54)		0.80 (0.52)		0.90 (0.54)	
CI	(0.81, 0.91)		(0.73, 0.87)		(0.85, 0.95)	
**Mean (SD) (from actual score) (11 question with max. 3 score each = 33 score)**	10.36 (6.43)		9.65 (6.27)		10.80 (6.54)	
**CI**	(9.8, 10.91)		(8.77, 10.52)		(10.1, 11.5)	

HAQ – Health Assessment Questionnaire, SD – standard deviation, CI – confidence interval

### Disability due to pain in extremities according to gender

**Figure 2:**
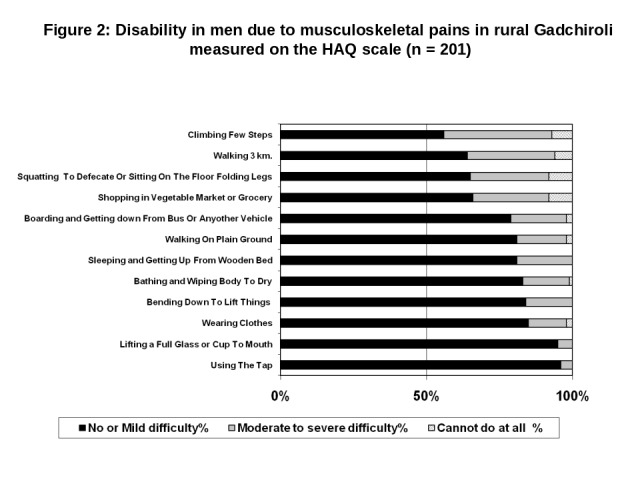
Disability in men due to musculoskeletal pains in rural Gadchiroli measured on the HAQ scale (n = 201). HAQ – Health Assessment Questionnaire.

**Figure 3:**
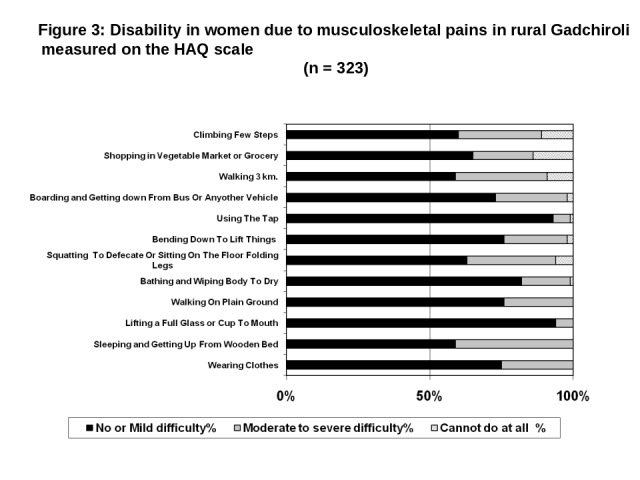
Disability in women due to musculoskeletal pains in rural Gadchiroli measured on the HAQ scale (n = 323). HAQ – Health Assessment Questionnaire.

The gender specific disability score showed higher burden of disability in women compared to men (**Table 3**, Tables S1 and S2 in the **Online Supplementary Document**, and **Figure 2** and **Figure 3**). Overall, no difficulty (zero), mild difficulty (>zero and ≤1), moderate to severe difficulty (>1 and ≤1.5) and complete inability (>1.5) to do an activity was identified in 2%, 68%, 19% and 11% of the men participants respectively and 1%, 67%, 17% and 16% women participants respectively.

### Activity limitation due to back pain in men

Of the 241 men participants, a significant 47% had no difficulty in getting sound sleep, whereas only 11% had no difficulty in agrarian work. Mild difficulty was faced by 46% and 42% in sitting and standing for long time respectively. Significant proportion of participants (60%) faced moderate to severe difficulty in agrarian work, followed by lifting heavy things or bending (42%). These two activities were also represented by the maximum participants in the category ‘cannot do at all’, with 11% each (**Table 4**).

**Table 4:** Activity limitation due to back pain in men in rural Gadchiroli (n = 245)

Activity	No difficulty%	Mild difficulty%	Moderate to severe difficulty %	Cannot do at all %
	**0**	**1**	**2**	**3**
Agrarian tasks (ploughing, harvesting)	11	19	60	11
Cutting wood	16	33	43	8
Lifting heavy things by bending or lifting and carrying on head	16	31	42	11
Squatting or getting up after defecation	39	38	21	3
Travelling by bus	43	39	18	0
Sitting upright/straight	31	41	27	1
Standing for long time	34	42	21	3
Sitting for long time	24	46	30	1
Regular walking	36	37	24	2
Walking farmlands or climbing up riverbeds (pains and need support of a stick or a person)	20	33	40	7
Sound sleep	47	32	20	1

### Activity limitation due to back pain in women

Of the 471 women participants, a significant 47% had no difficulty in getting sound sleep. Mild difficulty was faced by 43% and 40% in sitting for long time and regular walking and standing for long time, respectively. A large proportion of participants faced moderate to severe difficulty in household (69%) and agrarian tasks (64%). 12% of the participants were completely unable to perform agrarian tasks while 10% were unable to lift heavy loads by bending or carrying on head (**Table 5**).

**Table 5:** Activity limitation due to back pain in women in rural Gadchiroli (n = 471)

Activity	No difficulty %	Mild difficulty%	Moderate to severe difficulty%	Cannot do at all %
	**0**	**1**	**2**	**3**
Household tasks (sweeping, cooking, washing clothes)	6	20	69	4
Drawing water from well/bore well	21	34	39	6
Agrarian tasks (sowing, harvesting, cutting of paddy or grass)	8	15	64	12
Sitting upright/straight	20	33	46	1
Lifting heavy things by bending or carrying on head (wood logs/grass/water pots)	11	37	42	10
Squatting and getting up after defecation	38	30	31	1
Standing for long time	24	38	36	3
Sitting for long time	18	43	38	1
Regular walking	24	40	33	3
Walking farmlands or climbing up riverbeds, (pains and need support of stick or person)	11	38	42	9
Sound sleep	47	35	17	1

## DISCUSSION

Overall, we found that though a significant proportion of community suffers from PBE, the nature of disability is mild in most of the individuals for most of the routine activities. For the first time, we also developed and used a culture specific questionnaire (RIALQ) for understanding the effect of BP on activities of daily living in the local rural community.

We observed that despite significant period prevalence of BP (76%) and musculoskeletal pains (71%) [13], relatively less participants were completely unable to do an activity. For example, only 11% of men and 12% of women with back pain were absolutely unable to do agrarian tasks. Similarly, the proportion of participants with pain in extremities, who were completely unable to perform an activity was less than 10% for most of the activities. This finding is similar to another study in rural Indian setting [12]. Interestingly these findings with respect to disability also corroborate with the intensity of pain, which was mostly mild (81%) suggesting that majority of the participants with PBE are suffering with mild pain and disability. This underlines need to identify or develop appropriate community based interventions which may allow earlier return to work through coping up and identifying alternative activities, so as to allow uninterrupted daily living.. However more significant activity limitation in agrarian tasks is worrisome as in an agrarian community livelihood is dependent on manual labor and indicates need to develop intervention to reduce higher frequency of moderate to severe disability among those with pain in back. Possible interventions to reduce this disability could be use of pain relieving agents and community-based rehabilitation for postural corrections, muscle strengthening. Given the high prevalence of PBE, the interventions to reduce disability due to PBE needs to be available at community level.

To the best of our knowledge, this is the first study from rural India reporting disability due to PBE a community that is largely agrarian and involved in manual labour. Other studies were from communities with less preponderance of manual agrarian labour [12].

The study had several strengths. The two study villages were randomly selected from a list of villages after excluding atypical (too large, too small, peri-urban villages with a primary health centre) villages. The participation rate of the adults in the villages was high. The data collection was done by CHWs with more than 15 years of experience and was done using a systematically developed rural activity-specific questionnaire and HAQ.The possibility of incomplete or inaccurate data collection was minimized by rigorous training of CHWs in using the questionnaires, rigorous supervision and quality checks during data entry.

A key limitation of the study was the possibility of recall loss by the participants which would probably underestimate rather than overestimate the disability. The villages where the study was conducted had male and female CHWs who routinely provided subsidized treatment for pains with tablet aspirin for the past 20 years. This can also reduce duration and intensity of pain and the disability. On the other hand, we also cannot rule out that some of the participants may have overstated the disability.

## CONCLUSION

In conclusion, this population-based study in rural Gadchiroli demonstrates predominantly mild to moderate disability due to PBE, except for agrarian tasks for men and women and household tasks for women where more than 60% individuals with PBE reported moderate to severe disability. The higher frequency of disability in performing agrarian tasks calls for interventions to reduce this disability as the livelihood of the population is dependent on agrarian tasks. The study also employs for the first time an indigenously developed questionnaire to identify activity limitation due to back pain in rural, agrarian areas.

## Additional material


Online Supplementary Document
