# Supplementary material for: Activity limitation and disability due to pain in back and extremities in rural population: A community-based study during a period of twelve months in rural Gadchiroli, India
Source: J Glob Health. 2021 Nov 27;11:12003. doi: 10.7189/jogh.11.12003 (PMC8645241; doi:10.7189/jogh.11.12003)
Supplement: Online Supplementary Document [file jogh-11-12003-s001.pdf]

Table S1: Disability in women due to musculoskeletal pains in rural Gadchiroli measured on the HAQ* scale

(n = 323)

Question	No difficulty%	Mild difficulty%	Moderate to severe difficulty %	Cannot do at all %
	**0**	**1**	**2**	**3**
Wearing Clothes	48	27	24	0
Sleeping and Getting Up From Wooden Bed	18	41	41	0
Lifting a Full Glass or Cup To Mouth	74	20	6	0
Walking On Plain Ground	37	38	24	0
Bathing and Wiping Boby To Dry	52	31	17	1
Sitting To Defecate Or On The Floor Folding Legs	28	36	31	6
Bending Down To Lift Things	34	43	22	1
Using The Tap	80	13	6	1
Boarding and Getting down From Bus Or Anyother Vehicle	32	42	25	2
Walking 3 km.	22	37	32	9
Shopping in Vegetable Market or Grocery	39	26	21	14
Climbing Few Steps	25	34	29	11

* HAQ: Health Assessment Questionnaire

Table S2: Disability in men due to musculoskeletal pains in rural Gadchiroli measured on the HAQ* scale

(n = 201)

**Question**	**No difficulty%**	**Mild difficulty%**	**Moderate to severe difficulty %**	**Cannot do at all %**
	**0**	**1**	**2**	**3**
Wearing Clothes	61	24	13	1
Sleeping and Getting Up From Wooden Bed	31	49	19	0
Lifting a Full Glass or Cup To Mouth	76	19	5	0
Walking On Plain Ground	48	32	17	2
Bathing and Wiping Boby To Dry	51	32	16	1
Sitting To Defecate Or On The Floor Folding Legs	24	41	27	7
Bending Down To Lift Things	50	33	16	0
Using The Tap	80	16	4	0
Boarding and Getting down From Bus Or Anyother Vehicle	38	41	19	1
Walking 3 km.	22	41	30	6
Shopping in Vegetable Market or Grocery	33	33	26	8
Climbing Few Steps	22	33	37	7

* HAQ: Health Assessment Questionnaire

Table S3: HAQ Questionnaire

^^SEARCH**

Form No:

Ask the following questions if the respondent has pain in hands/legs/joints/limbs

Sr. No	Daily Activities				
	**Are you able to do the followings?**	**Able to do without any problem**	**A little problem while doing**	**More problem while doing**	**Not able to do**
	**Score**	**0**	**1**	**2**	**3**
**1**	Wearing own clothes				
**2**	Sleeping on bed and getting up				
**3**	Lifting a full glass or cup to mouth				
**4**	Walking on plain land				
**5**	Taking bath and wiping the body				
**6**	Squatting to defecate Or sitting on the floor folding legs				
**7**	Bending down to lift things				
**8**	Opening and stopping the tap				
**9**	Boarding or getting down from bus or any vehicle				
**10**	Walking 3 KM.				
**11**	Buying goods from shops or vegetable markets				
**12**	Climbing few steps				

Maximum score = 36

No. of incorrect entries in the form =

Obtained score =

Percentage =

Table S4: RIALQ to measure activity limitation due to back pain in men

^^SEARCH**

Form No:

Male

Sr. No	Activities males cannot do due to back pain	No	A little	Medium	Too much	Disabled
1	Do you face difficulties in farming tasks (ploughing, harvesting) due to back pain?					
2	Do you face difficulties in cutting wood due to back pain?					
3	Do you face difficulties travelling by bus due to back pain?					
4	Do you face difficulties in lifting by bending and lifting and carrying heavy things on head due to back pain?					
5	Do you face difficulties in squatting for toilet and getting up after due to back pain?					
6	Do you face difficulties in sitting straight or upright due to back pain?					
7	Do you face difficulties in sitting for a long time due to back pain?					
8	Do you face difficulties in standing for a long time due to back pain?					
9	Do you have problems in regular walking due to back pain? (Unable to walk as before or as usual)					
10	Di you feel it is difficult to move inside of pond or riverbed or walk across the farm land due to back pain? Do you need support of stick or other person during such?					
11	Do you sleep well in presence of back pain?					
	**Total**					

A little : Has pain but is able to works as usual
Medium: Has pain, and can only partially work
Too Much : Has pain and unable to any work at all.
Disabled : Has to only sleeps / lie down due to pain.

Table S5: RIALQ to measure activity limitation due to back pain in women

^^SEARCH**

Form No:

Female

Sr. No	Activities males cannot do due to back pain	No	A little	Medium	Too much	Disabled
1	Do you have trouble in doing the household works (sweeping, cooking or washing clothes) due to back pain?					
2	Do you have difficulties in drawing water from well or bore well?					
3	Do you have difficulties in doing works of farming (sowing, cutting paddy or grass) due to back pain?					
4	Do you have difficulties in lifting heavy things as wood logs, grass or water pots by bending or carrying on head due to back pain?					
5	Do you face difficulties in sitting upright or straight due t back pain?					
6	Do you face difficulties in squatting for toilet and getting up due to back pain?					
7	Do you face difficulties in sitting for a long time due to back pain?					
8	Do you face difficulties in standing for a long time due to back pain?					
9	Do you have problems in regular walking due to back pain? (Unable to walk as before or as usual)					
10	Do you feel that back pain makes it difficult to move inside of pond, riverbed or walking farm land? Do you need support of stick or other person while moving in these areas?					
11	Do you sleep well in presence of back pain?					
	**Total**					

A little : Has pain but is able to works as usual
Medium: Has pain, and can only partially work
Too Much : Has pain and unable to any work at all.
Disabled : Has to only sleeps / lie down due to pain.
